# Supplementary material for: The Bacterial Amyloids Phenol Soluble Modulins from Staphylococcus aureus Catalyze Alpha-Synuclein Aggregation
Source: Int J Mol Sci. 2021 Oct 27;22(21):11594. doi: 10.3390/ijms222111594 (PMC8584152; doi:10.3390/ijms222111594)
Supplement: Supplementary file 1 [file ijms-22-11594-s001.zip › ijms-1409207-supplementary.pdf]

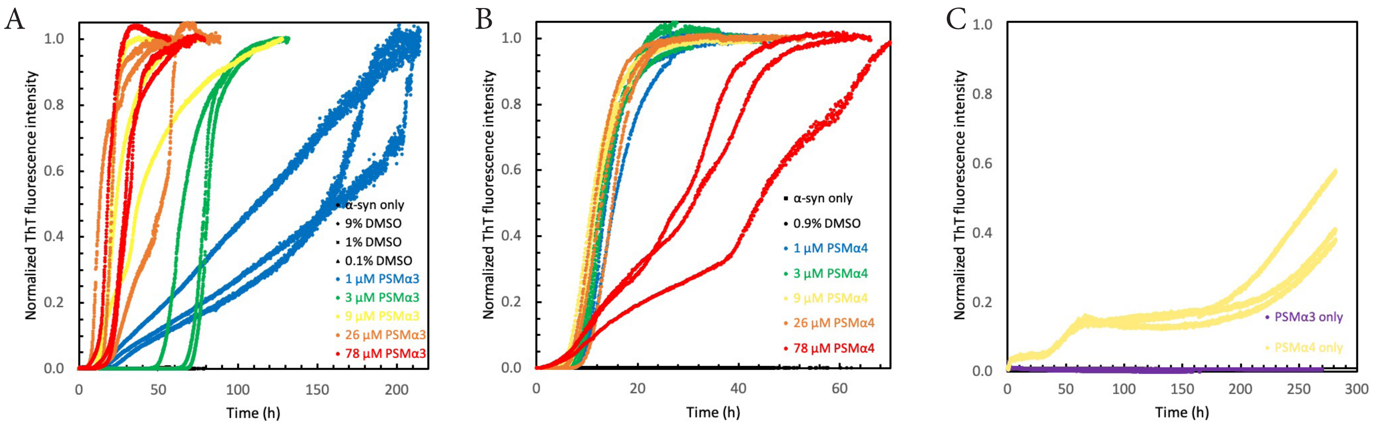


**Supplementary Figure S1. Aggregation of α-syn in the presence of PSMα peptides.** A, B. Normalized ThT fluorescence intensity as a function of time for 25 µM α-syn in the presence of varying PSMα3 (A) or PSMα4 (B) concentrations as given in the respective panel. C. Control experiments with only 78 µM PSMα3 (purple) or 78 µM PSMα4(yellow). All experiments started from monomers and were performed in 10 mM Tris, 50 mM NaCl, pH 7.6 at 37 °C under quiescent conditions.

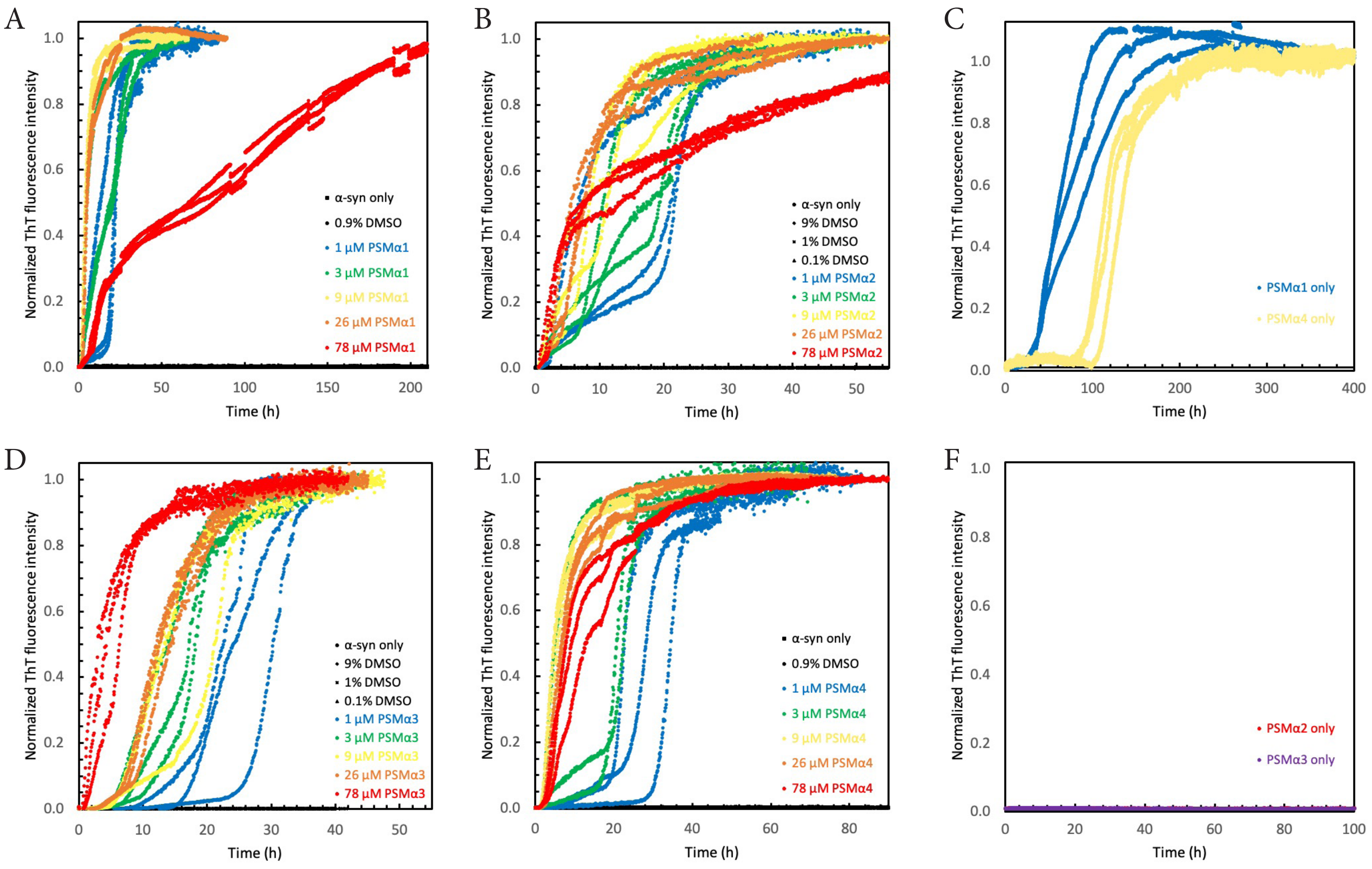


**Supplementary Figure S2. Aggregation of α-syn in the presence of PSMα peptides.** A, B, D, E. Normalized ThT fluorescence intensity as a function of time for 25 μM α-syn in the presence of varying PSMα1 (A), PSMα2 (B), PSMα3 (D), or PSMα4 (E) concentrations as given in the respective panel. C, F. Control experi-ments with only 78 μM PSMα1 (blue), PSMα4 (yellow), PSMα2 (red), or PSMα3 (purple). All experiments started from monomers and were performed in 10 mM MES, pH 5.5 at 37 °C under quiescent conditions.

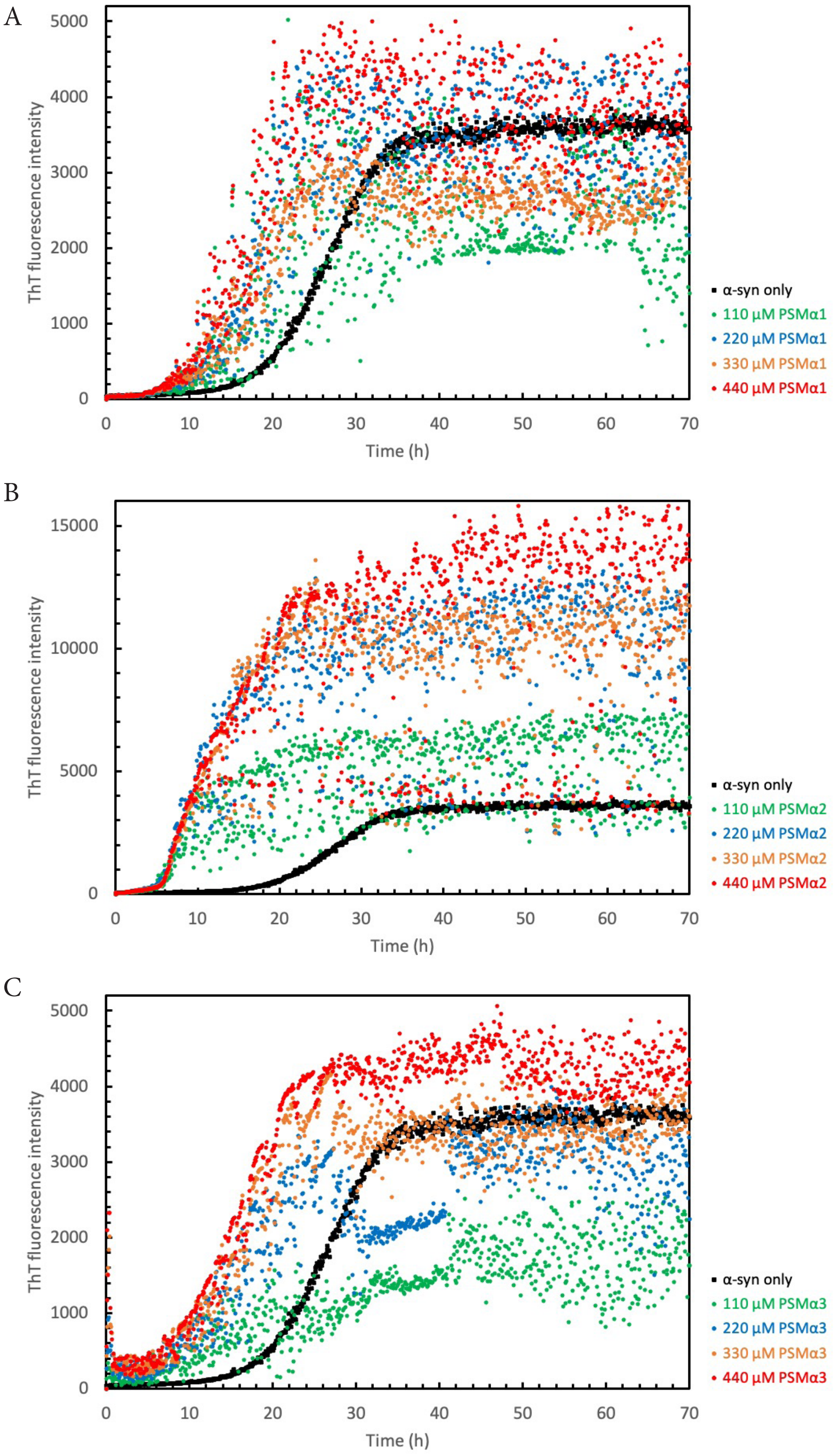


**Supplementary Figure S3. Aggregation of α-syn in the presence of PSMα peptides.** ThT fluorescence intensity as a function of time for 50 µM α-syn in the presence of varying PSMα1 (A), PSMα2 (B), or PSMα3 (C) concentrations as given in the respective panel. All experiments started from monomers and were performed in PBS at 37 °C under quiescent conditions in polystyrene plates. Each data point is an average of 2 replicates. PSMα only controls for these experiments can be found in reference 22.

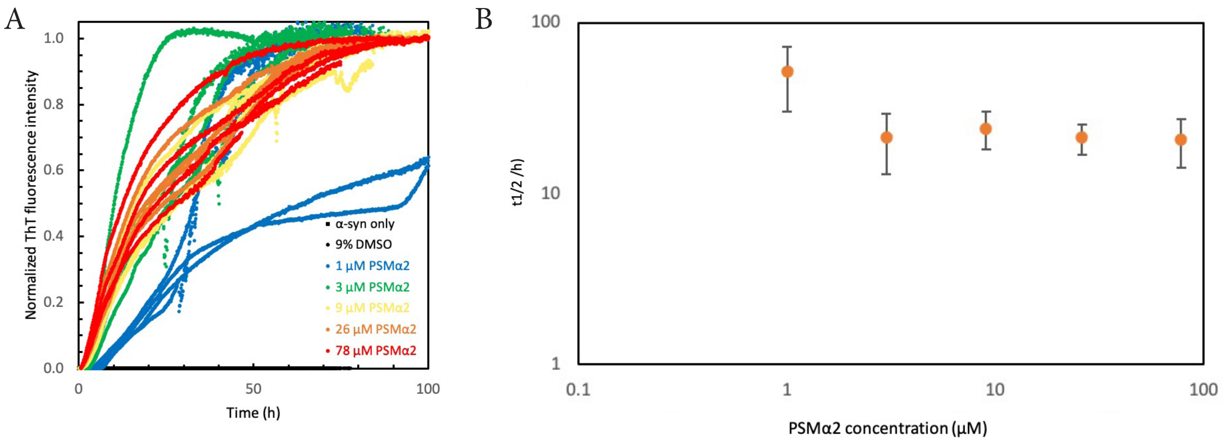


**Supplementary Figure S4. Aggregation of α-syn in the presence of PSMα2 peptide and excess DMSO.** A. Normalized ThT fluorescence intensity as a function of time for 25 μM α-syn aggre-gated in the presence of varying PSMα2 concentrations (μM) as given in the panel. All experi-ments started from monomers and were performed in 10 mM Tris, 50 mM NaCl, 9% DMSO, pH 7.6 at 37 °C under quiescent conditions. B. Plot of t1/2 as a function of PSMα2 concentration. The averages and standard deviations of 3-4 replicates are shown.

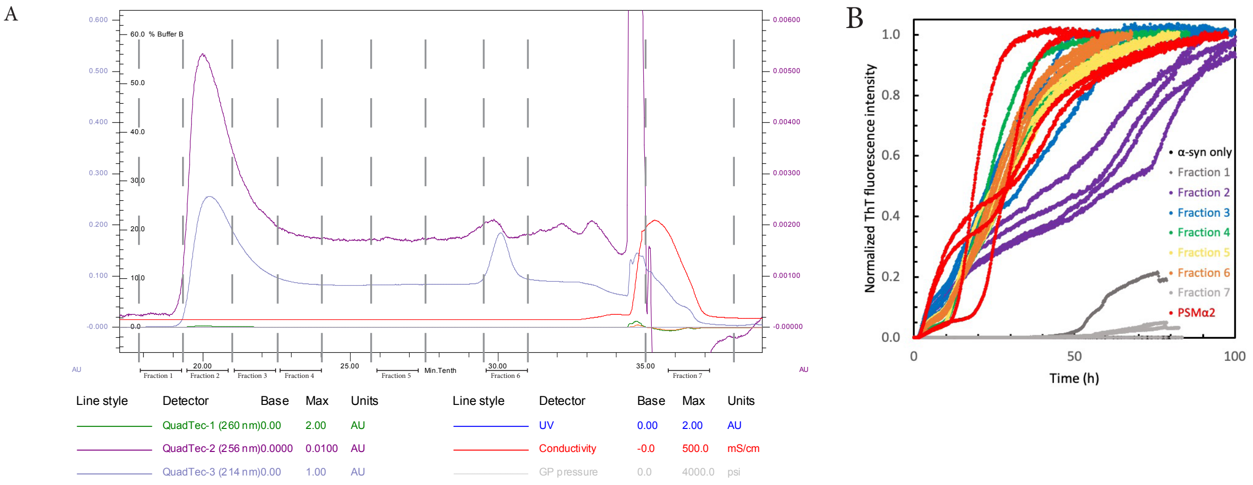


**Supplementary Figure S5. Aggregation of α-syn in the presence of FPLC-purified fractions of PSMα2 peptide.** PSMα2 was disolved in 6M GuHCl and purified into 10mM Tris, 50 mM NaCl, pH 7.6 using size exclusion chromatography. A. The absorbance at 214 and 256 nm was monitored and several fractions collected as indicated on the chromatogram. B. The normalized ThT fluorescence intensity as a function of time for 25 µM α-syn aggregated in the presence of the different purified PSMα2 fractions or in the presence of 9 µM non-purified PSMα2 in 1.1% DMSO.

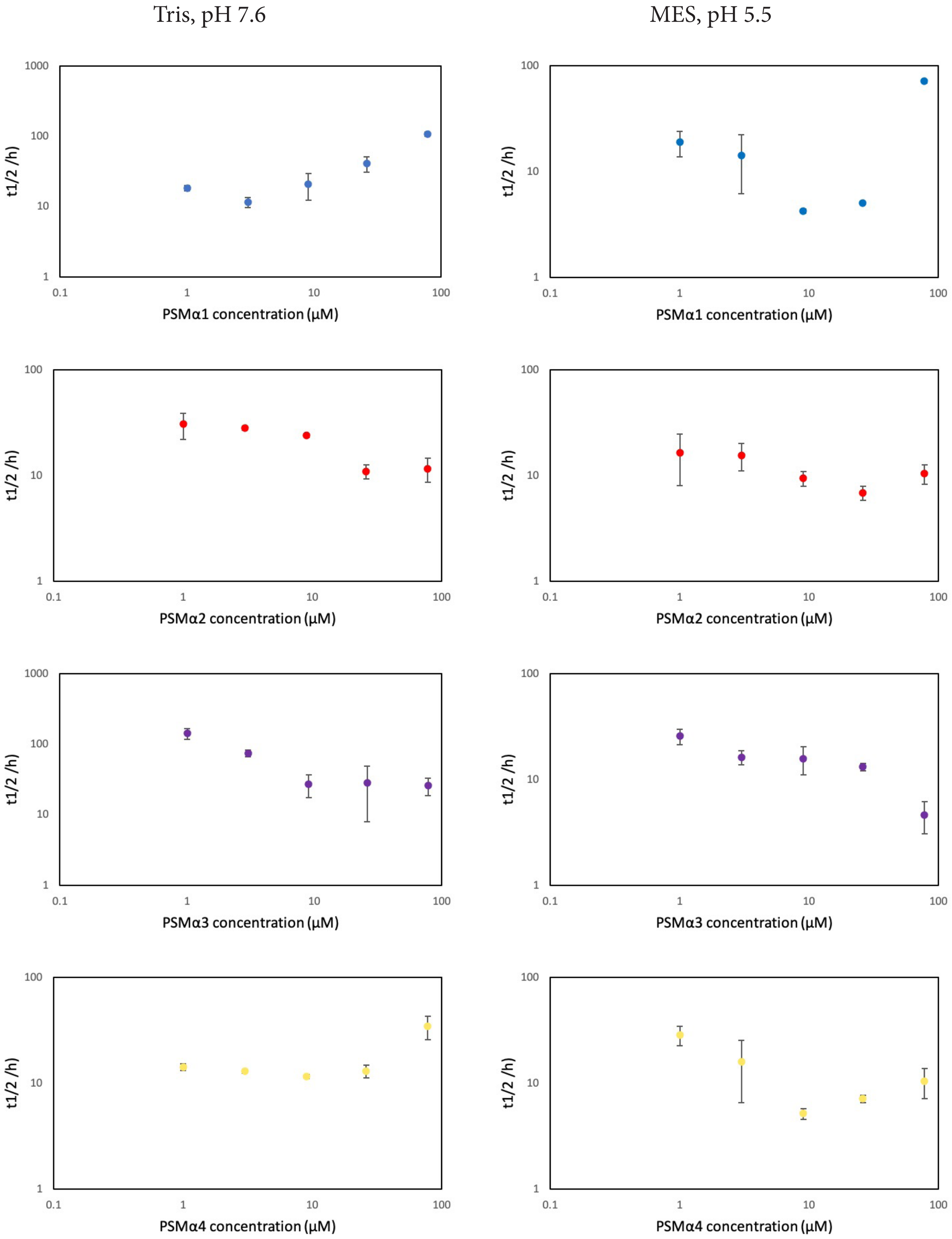


**Supplementary Figure S6. Aggregation of α-syn in the presence of PSMα peptide in neutral and slightly acidic buffers.** Plots of t1/2 of 25 μM α-syn aggregated in the presence of different concentrations of PSMα peptides as a function of peptide concentrations in 10 mM Tris, 50 mM NaCl, pH 7.6 or in 10 mM MES, pH 5.5. The averages and standard deviations of 3-4 replicates are shown except for 26 μM PSMα1 in MES buffer which only has 2 data points.

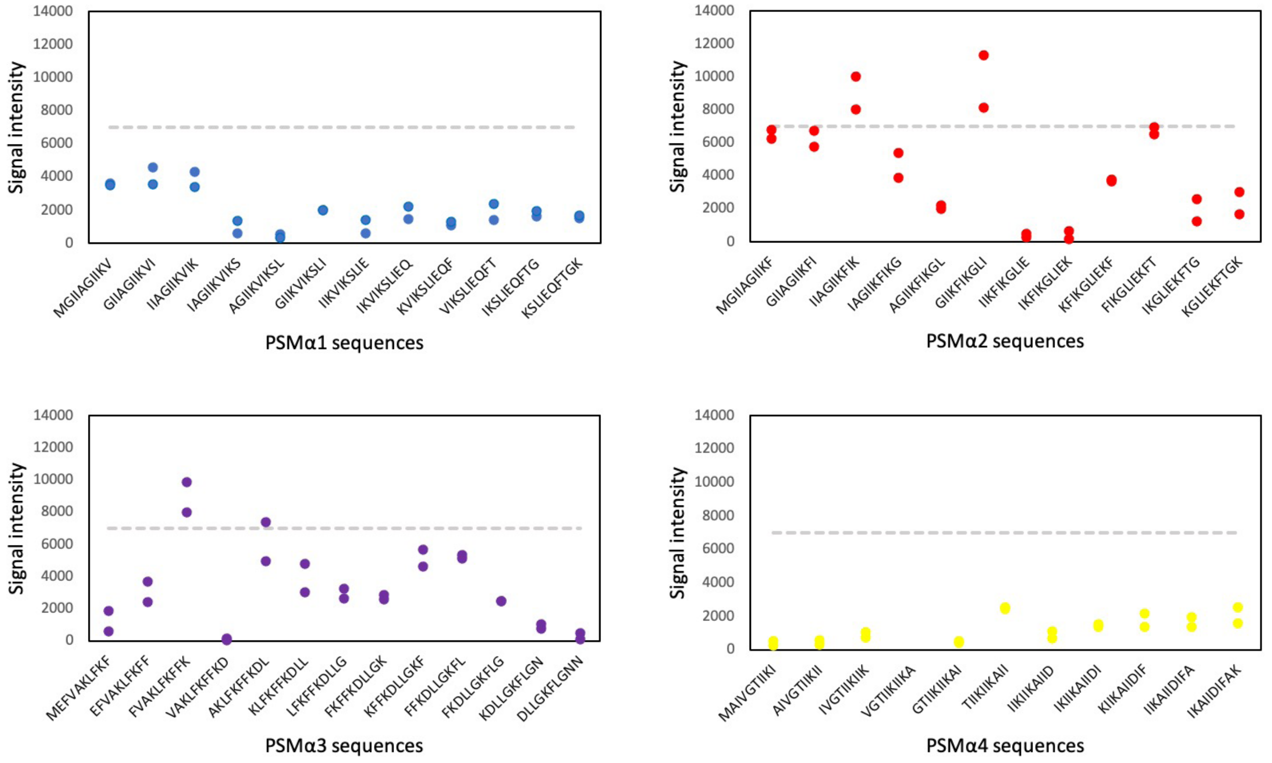


**Supplementary Figure S7. Arrays of PSMα sequences.** Alexa-fluor488-labeled-α-syn was added onto arrays coated with different 10-meric peptide sequences of each of the 4 studied PSMα peptides as per the figures. The signal intensity was measured and plotted for each sequence, with the dashed line at 7000 indicating the threshold value above which, α-syn was deemed to bidn to a peptide. Each experiment was performed twice.

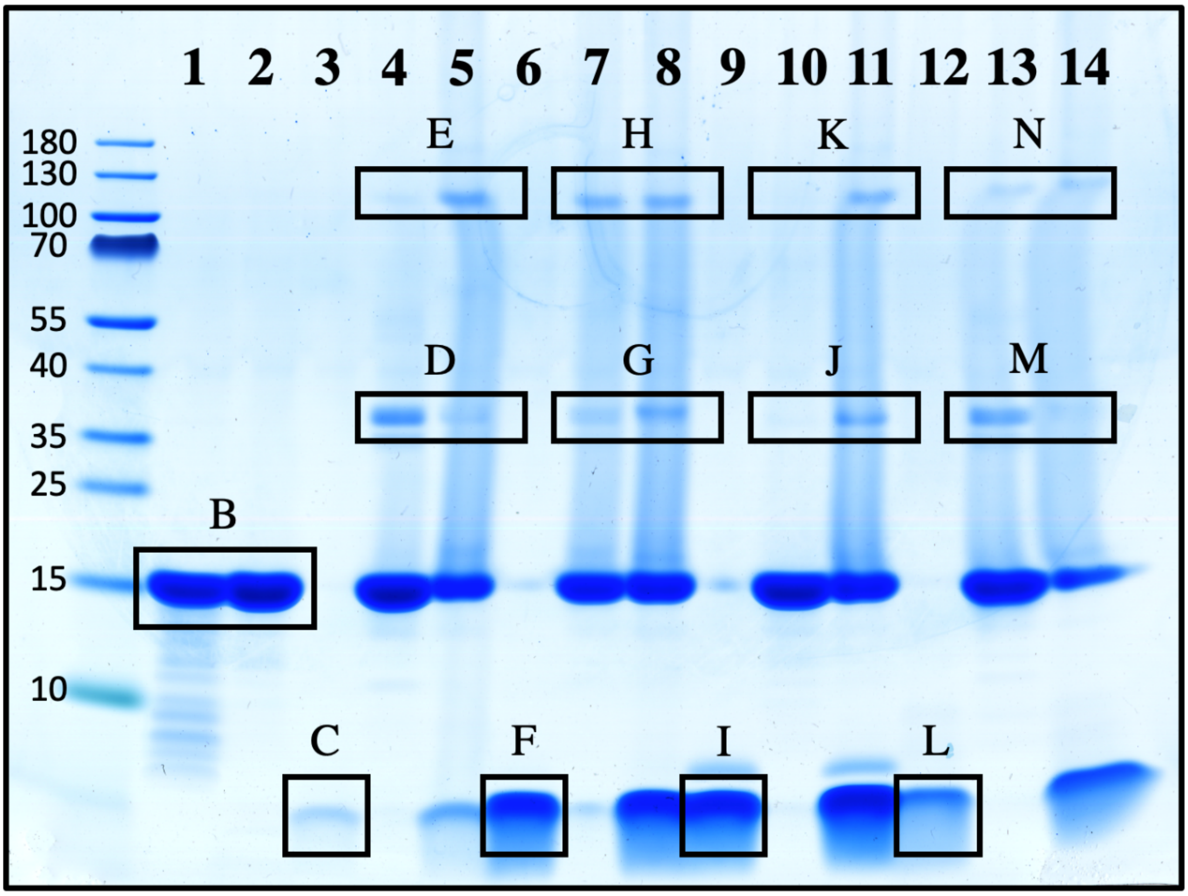


| Gel Lane | Sample | Gel band/Spectrum |
|---|---|---|
| | Std | |
| 1 | α-syn+ 0.9% DMSO | B |
| 2 | α-syn + 9% DMSO | |
| 3 | 77.7 µM PSMα1 | C |
| 4 | 25 µM α-syn + 0.97 µM PSMα1 | D and E |
| 5 | 25 µM α-syn + 77.7 µMPSMα1 | D and E |
| 6 | 77.7 µM PSMα2 | F |
| 7 | 25 µM α-syn + 0.97 µM PSMα2 | G and H |
| 8 | 25 µM α-syn + 77.7 µM PSMα2 | G and H |
| 9 | 77.7 µM PSMα3 | I |
| 10 | 25 µM α-syn + 0.97 µM PSMα3 | J and K |
| 11 | 25 µM α-syn + 77.7 µM PSMα3 | J and K |
| 12 | 77.7 µM PSMα4 | L |
| 13 | 25 µM α-syn + 0.97 µM PSMα4 1 | M and N |
| 14 | 25 µM α-syn + 77.7 µM PSM | M and N |

Gel band/ Spectrum B

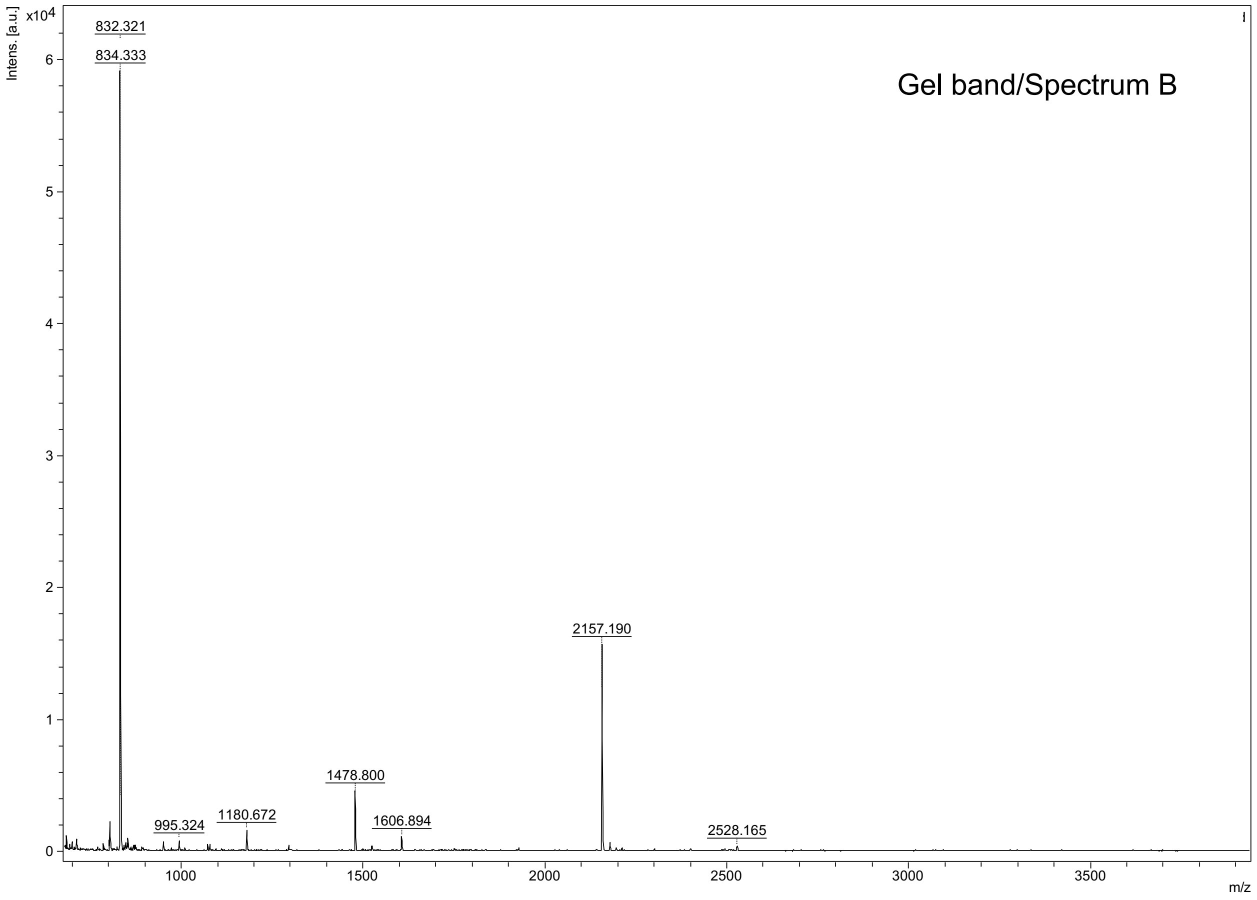

Gel band/ Spectra C, D and E (PSMα1)

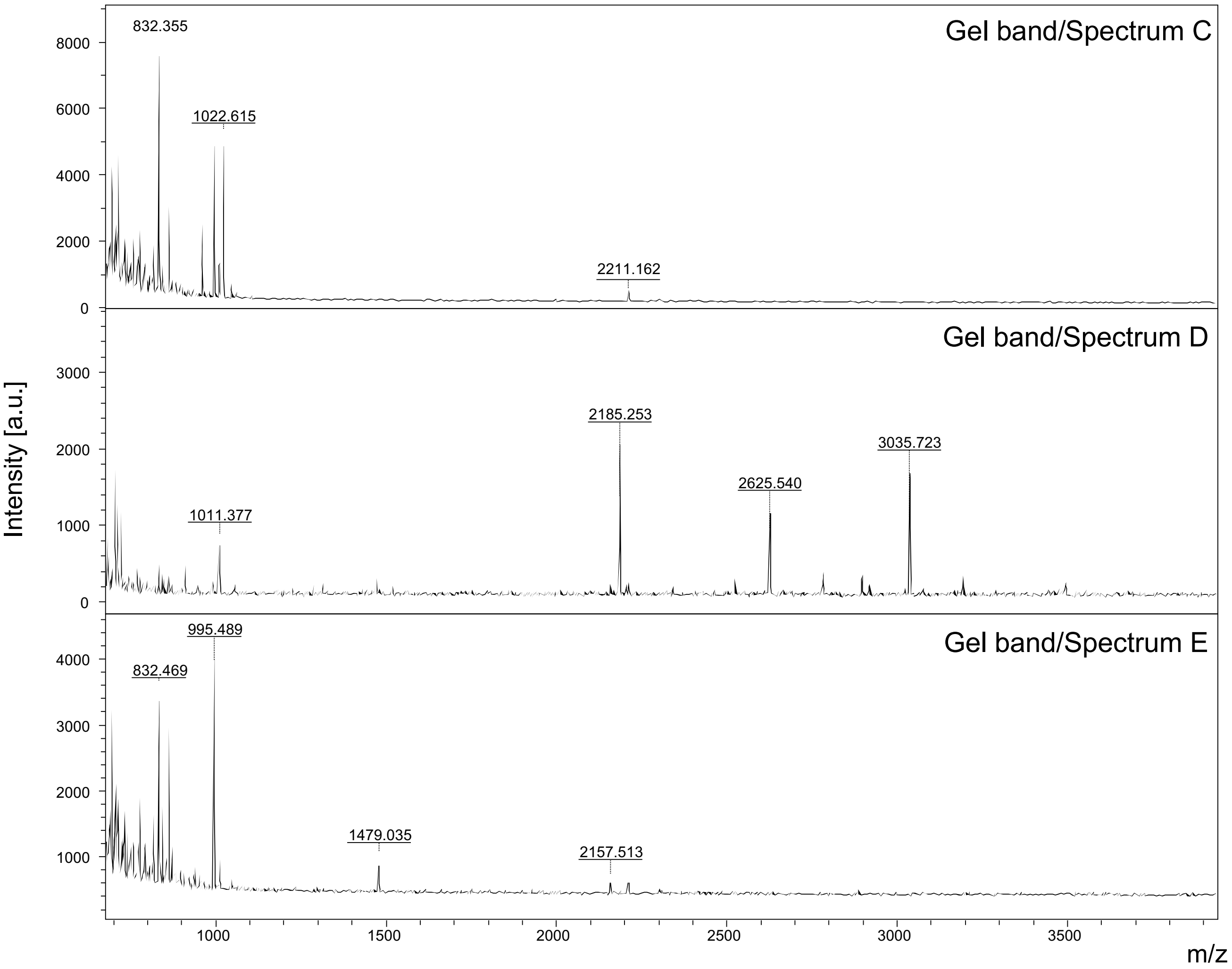


*MS spectra of in gel digested PSMα1 samples*. **Gel band/Spectrum: C** (2 kDa band), sample with 77.7 µM PSMα1; **D** (37 kDa band) and **E** (130 kDa band) sample with pooled digests of 25 µM α-syn + 0.97 µM PSMα1 and 25 µM α-syn + 77.7 µM PSMα1. The PSMα1 peptide SLIEQFTGK (aa from 13 to 21) with a theoretical mass of 1022.55 Da was detected in gel band C, (*see zoom on peptide in figure below)*

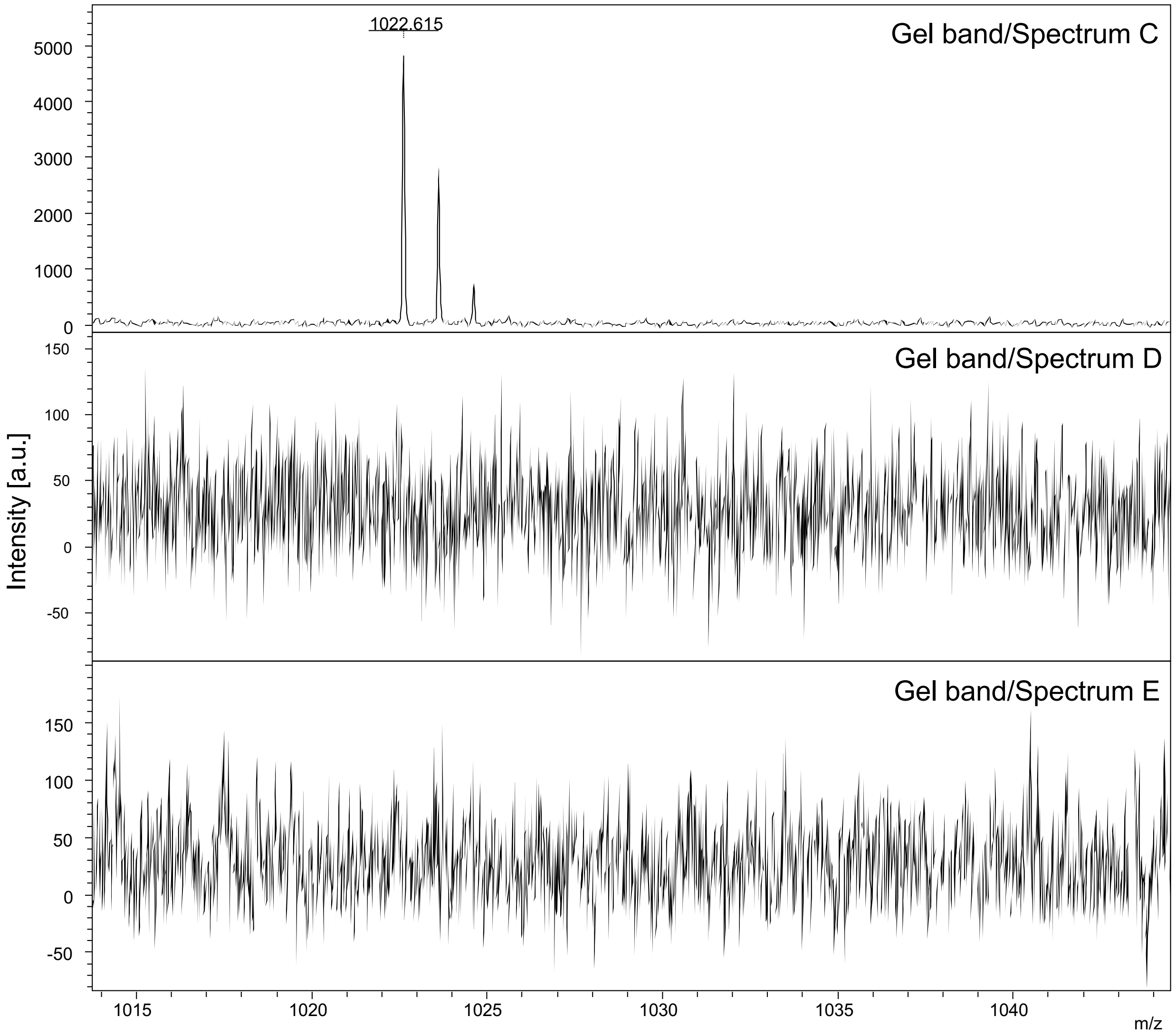
1022.615
Gel band/Spectrum C
5000
4000
3000
2000
1000
0
Gel band/Spectrum D
150
100
50
0
-50
Intensity [a.u.]
Gel band/Spectrum E
150
100
50
0
-50
1015
1020
1025
1030
1035
1040
m/z

Gel band/ Spectra F, G and H (PSMα2)

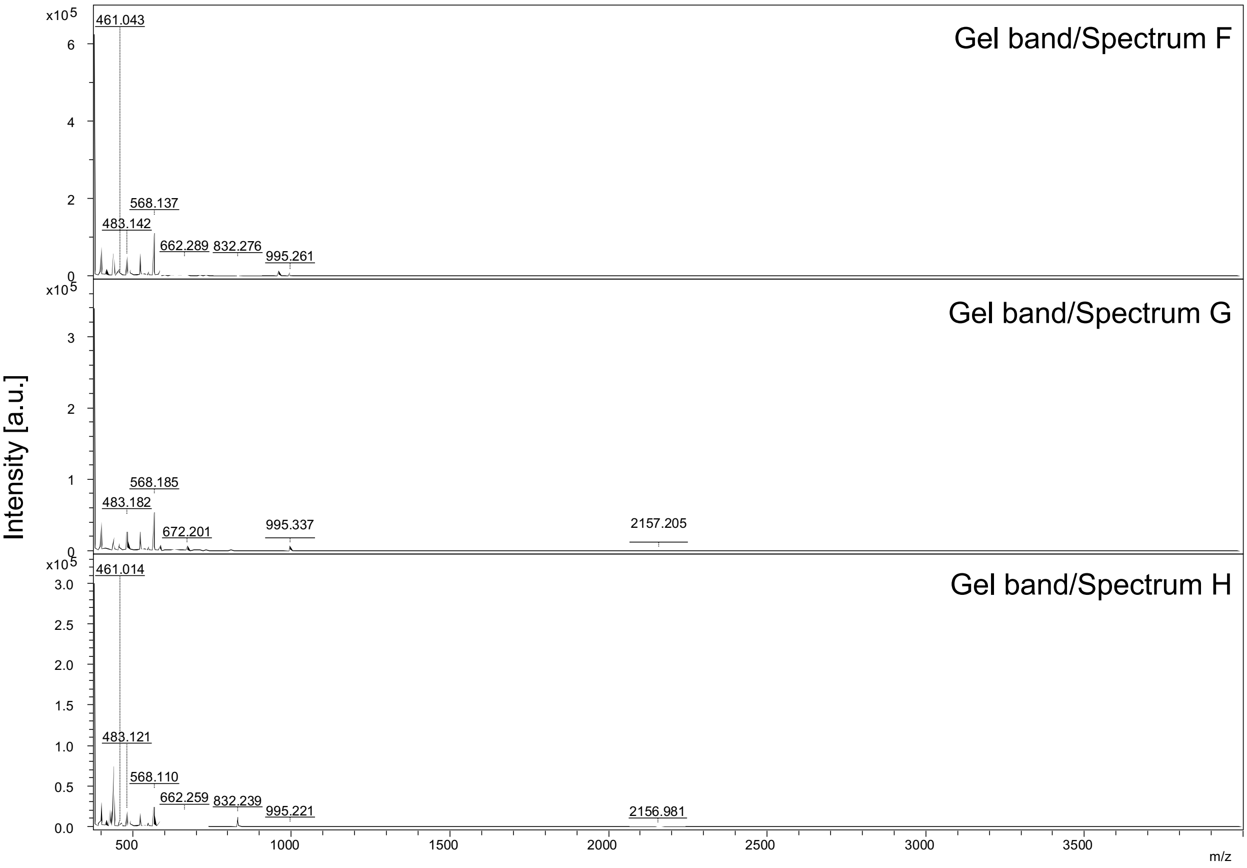


*MS spectra of in gel digested PSMα2 samples*. **Gel band/Spectrum: F** (2 kDa band), sample with 77.7 µM PSMα2; **G** (37 kDa band) and **H** (130 kDa band) sample with pooled digests of 25 µM α-syn + 0.97 µM PSMα2 and 25 µM α-syn + 77.7 µM PSMα2. The following PSMα2 peptides were detected and are presented in the zoomed spectra below: FIK (aa from 10 to 12) with a theoretical mass of 407.27 Da, FTGK (aa from 18 to 21) with a theoretical mass of 452.25 Da and GLIEK (aa from 13 to 17) with a theoretical mass of 559.34.27 Da were all detected in gel band F.

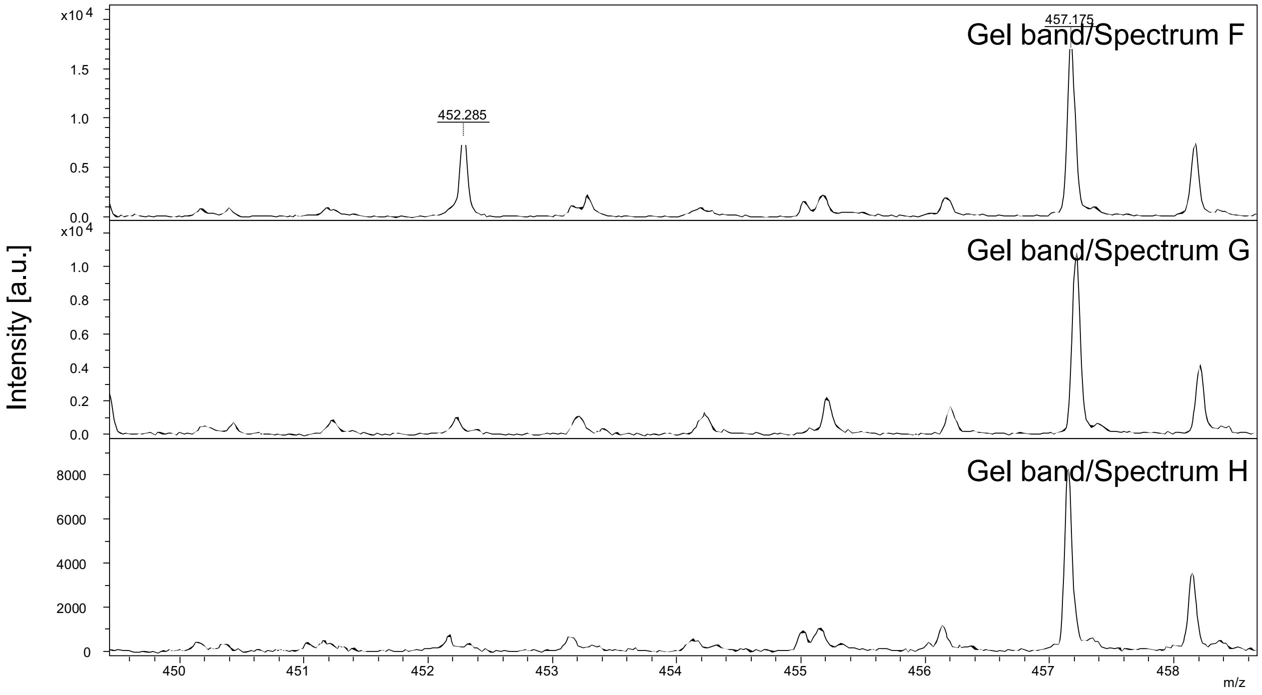

Gel band/Spectrum F
Gel band/Spectrum G
Gel band/Spectrum H
452.285
457.175
Intensity [a.u.]
m/z


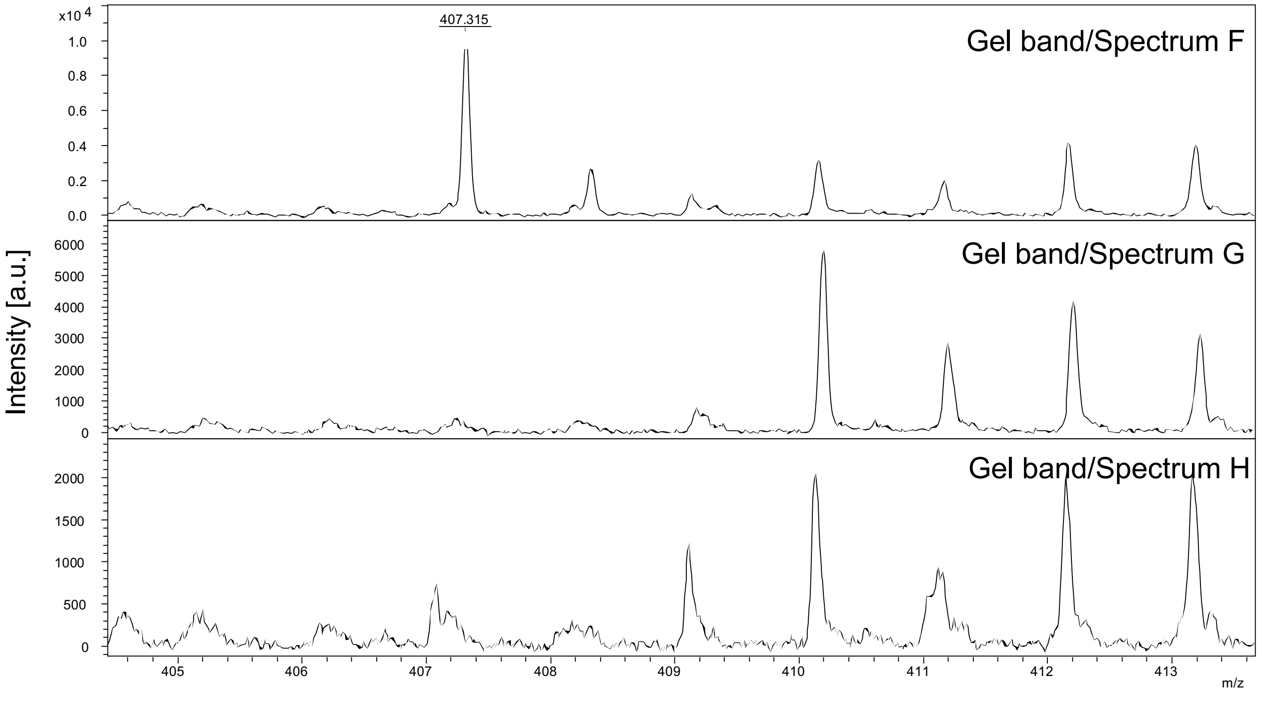

Gel band/Spectrum F
Gel band/Spectrum G
Gel band/Spectrum H
407.315
Intensity [a.u.]
m/z

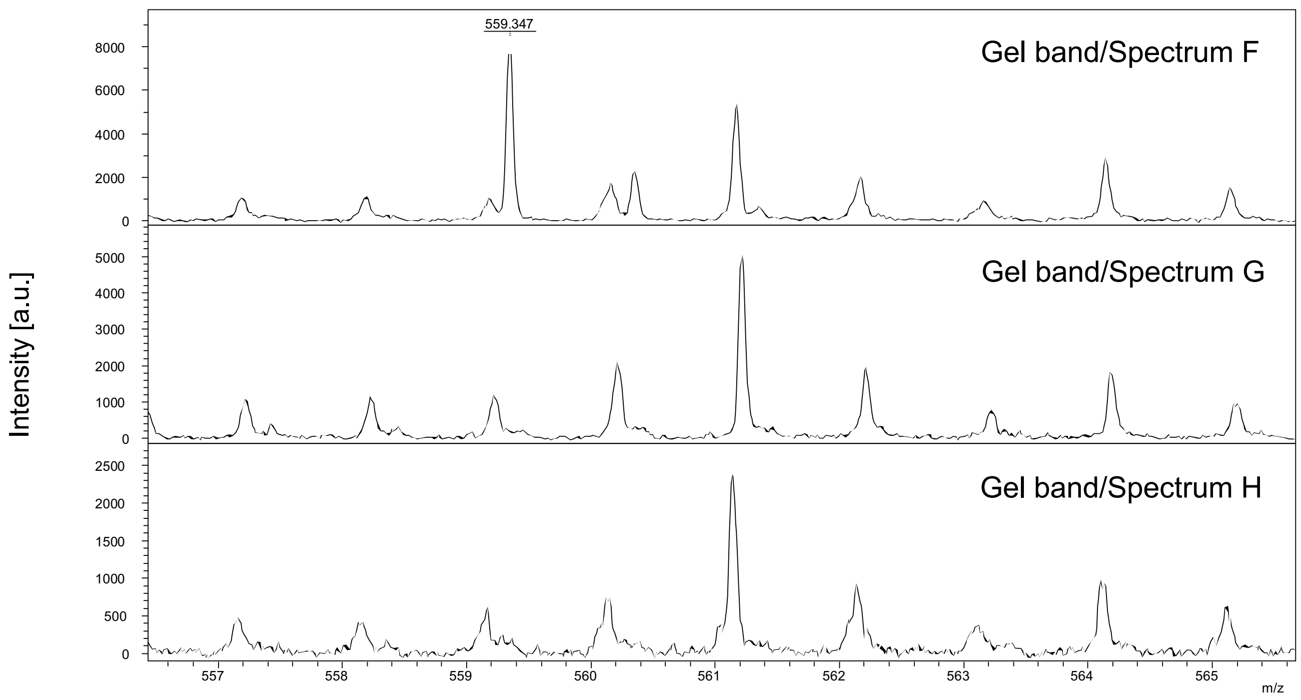
559.347
Gel band/Spectrum F
Gel band/Spectrum G
Gel band/Spectrum H
Intensity [a.u.]
m/z
557
558
559
560
561
562
563
564
565

Gel band/ Spectra I, J and K (PSMα3)

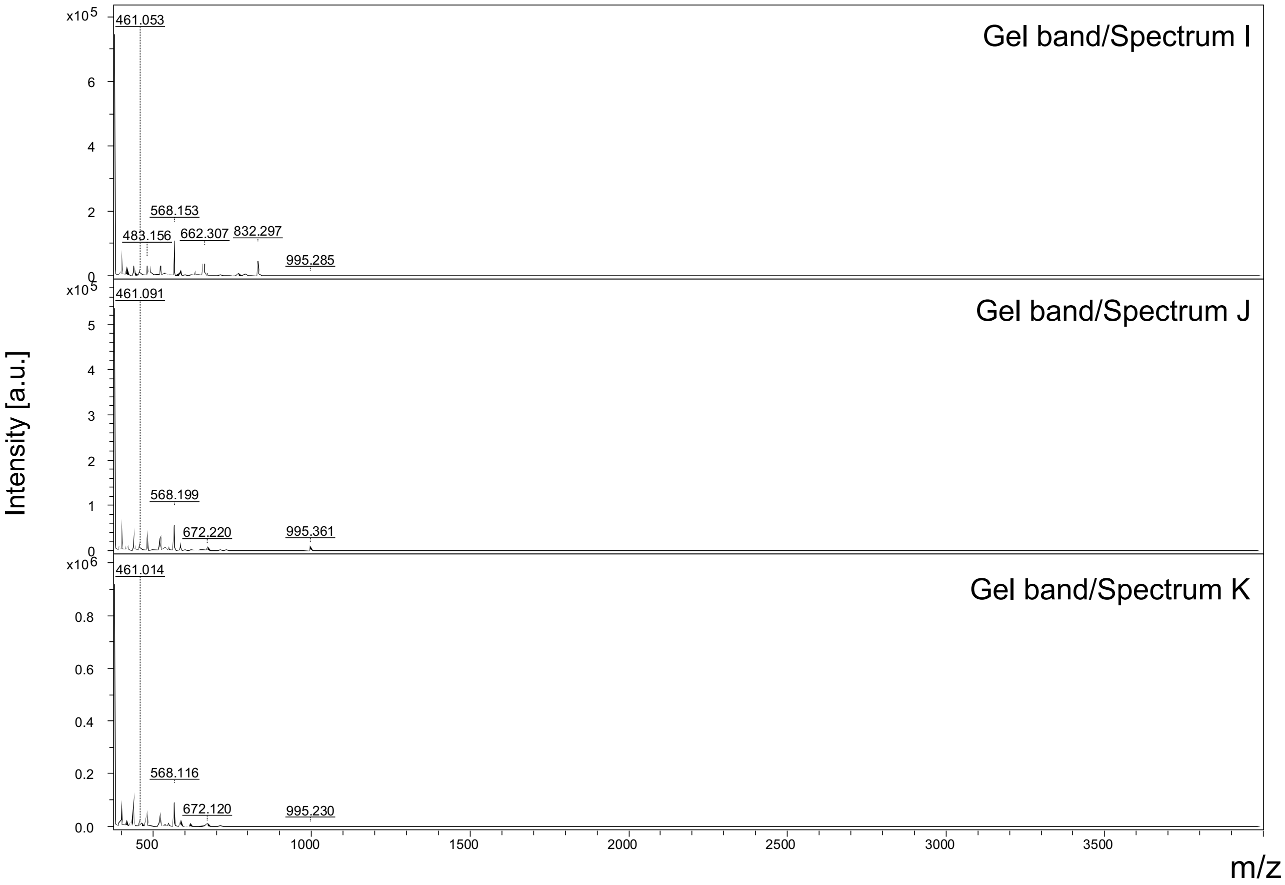


*MS spectra of in gel digested PSMα3 samples*. **Gel band/Spectrum: I** (2 kDa band), sample with 77.7 µM PSMα3; **J** (37 kDa band) and **K** (130 kDa band) sample with pooled digests of 25 µM α-syn + 0.97 µM PSMα3 and 25 µM α-syn + 77.7 µM PSMα3. The following PSMα3 peptides were detected and are presented in the zoomed spectra below: LFK (aa from 7 to 9) with a theoretical mass of 407.27 Da, MEFVAK (aa from 1 to 6) with a theoretical mass of 752.36 Da are both detected in gel band I.

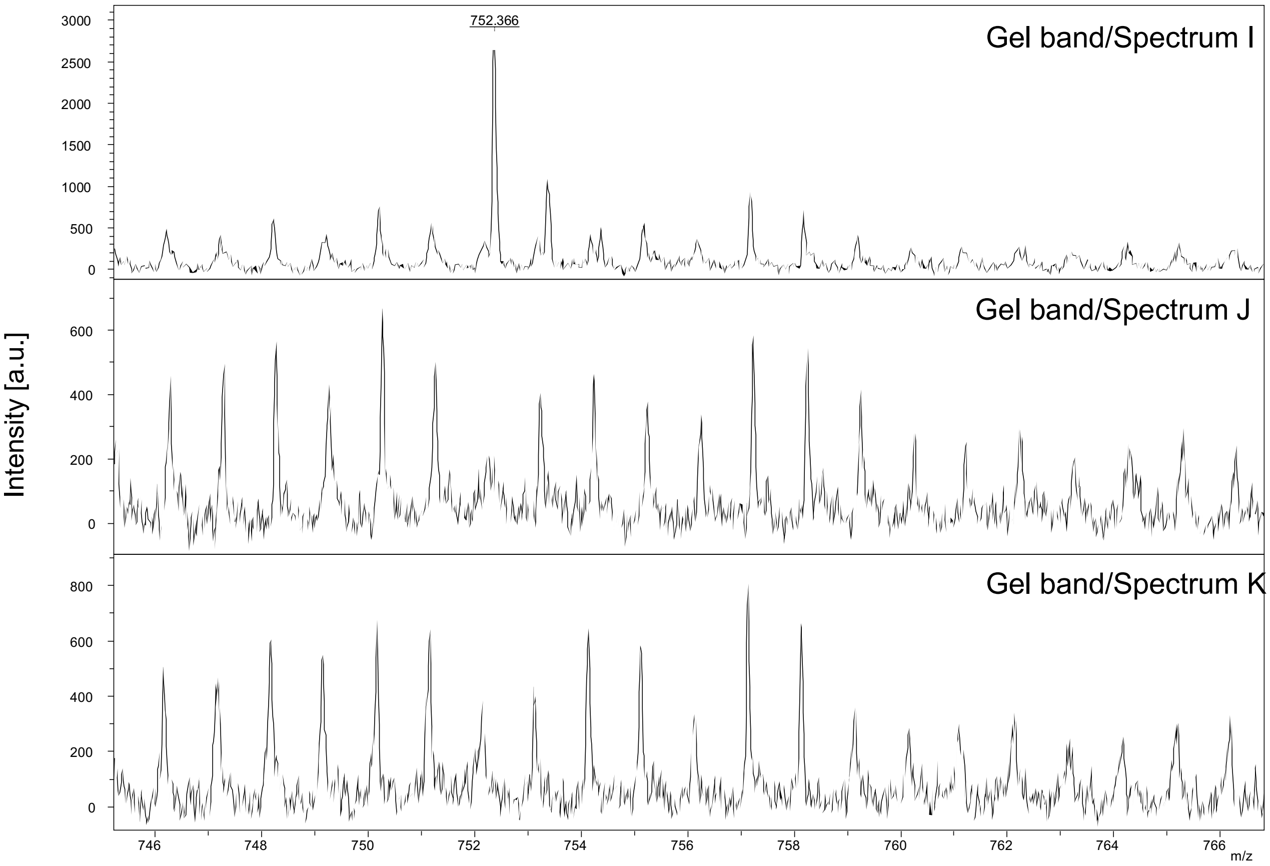
752.366
Gel band/Spectrum I
Gel band/Spectrum J
Gel band/Spectrum K
Intensity [a.u.]
m/z

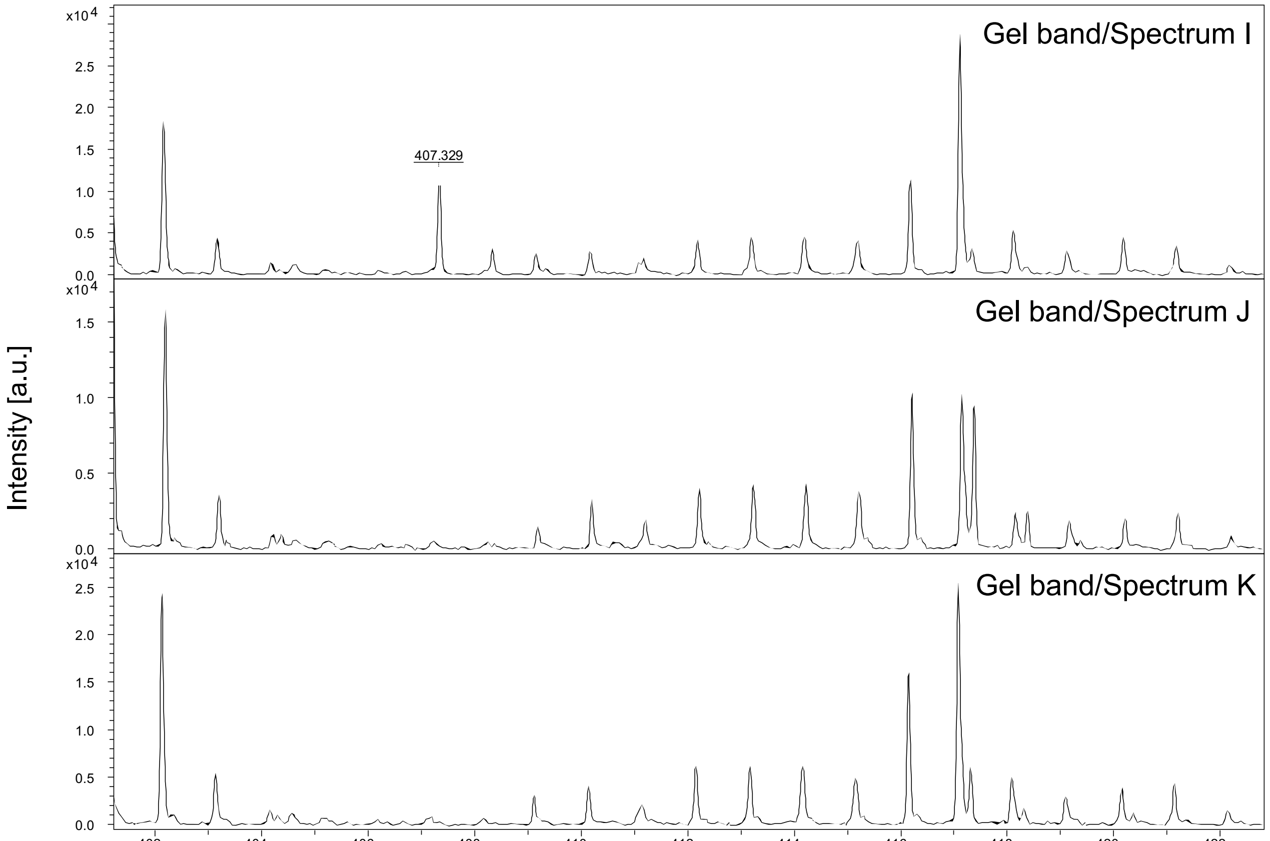
407.329
Gel band/Spectrum I
Gel band/Spectrum J
Gel band/Spectrum K
Intensity [a.u.]
x10^4
m/z

Gel band/ Spectra L, M and N (PSMα4)

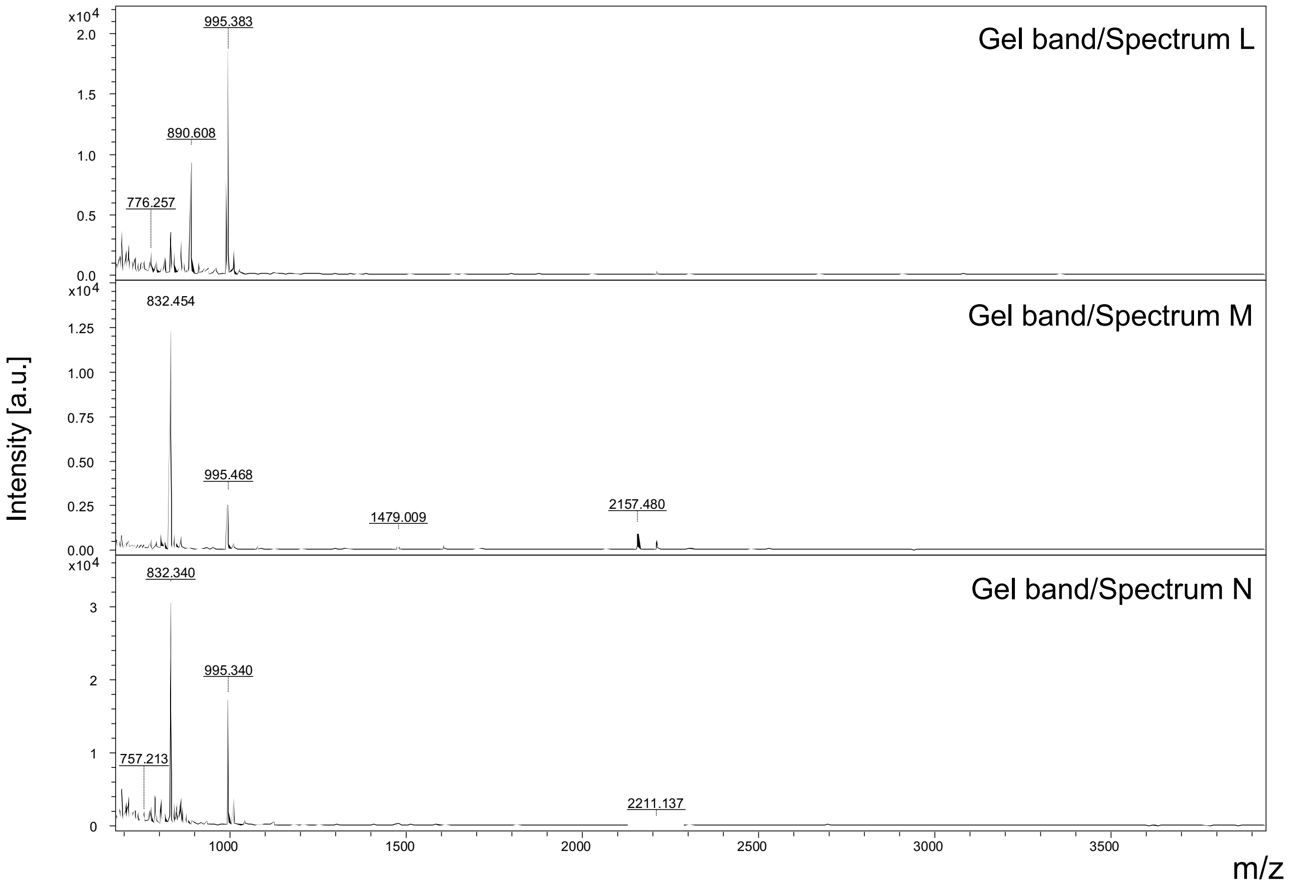


*MS spectra of in gel digested PSMα4 samples*. **Gel band/Spectrum: L** (2 kDa band), sample with 77.7 µM PSMα4; **M** (37 kDa band) and **N** (130 kDa band) sample with pooled digests of 25 µM α-syn + 0.97 µM PSMα4 and 25 µM α-syn + 77.7 µM PSMα4. The following PSMα4 peptide was detected in gel band L and is presented in the zoomed spectrum below: AIIDIFAK (aa from 13 to 20) with a theoretical mass of 890.53 Da.

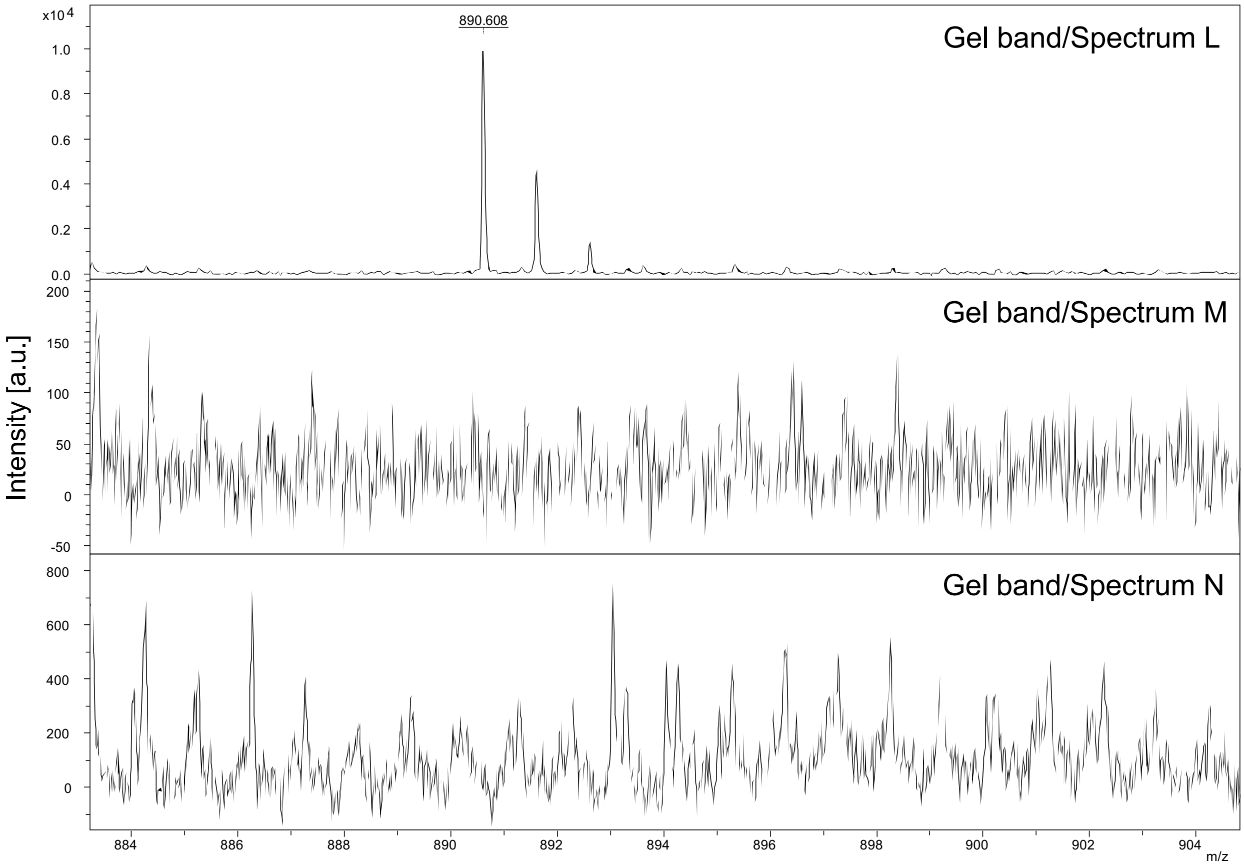


**Supplementary Figure S8. Mass Spectrometry analyses of aggregated α-syn and PSMα peptides.** A. Samples taken at the plateau of aggregation reactions of 25 µM α-syn in the absence or presence of PSMα (1-4) peptides and PSMα (1-4) peptides incubated without α-syn in 10 mM Tris, 50 mM NaCl, pH 7.6 were size separated on a Tricine SDS gel. The monomer α-syn bands, monomer PSMα peptide bands and the 37 kDa and 120 kDa bands (as indicated on the gel) were digested and analyzed by mass spectrometry. While the expected peptides of all the PSMα peptides were identified in the <10 kDa bands, only α-syn peptides were detected in the 37 and 120 kDa bands. B-N. The MS spectra of each of the digested bands.

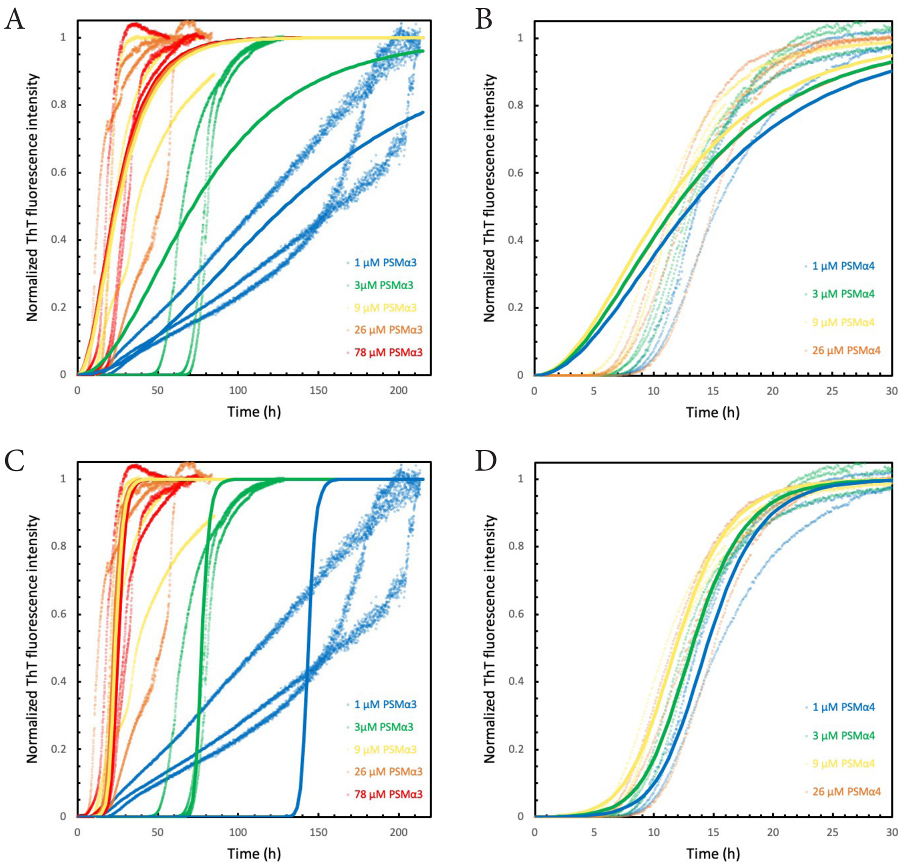


**Supplementary Figure S9. Fitting of models of nucleation and elongation to aggregation kinetics of α-syn in the presence of PSMα peptides.** The ThT fluorescence intensities as a function of time of 25 µM α-syn aggregation induced by varying PSMα3 (A, C) and PSMα4 (B, D) concentrations (µM)were fitted to models of primary nucleation and elongation (A, B) or to models of primary nucleation, elongation and secondary nucleation (C, D). The dotted lines are the normalized ThT fluorescence intensitites and the solid lines are the fits as calculated by Amylofit. All experiments started from monomers and were performed in 10 mM Tris, 50 mM NaCl, pH 7.6 at 37 °C under quiescent conditions.

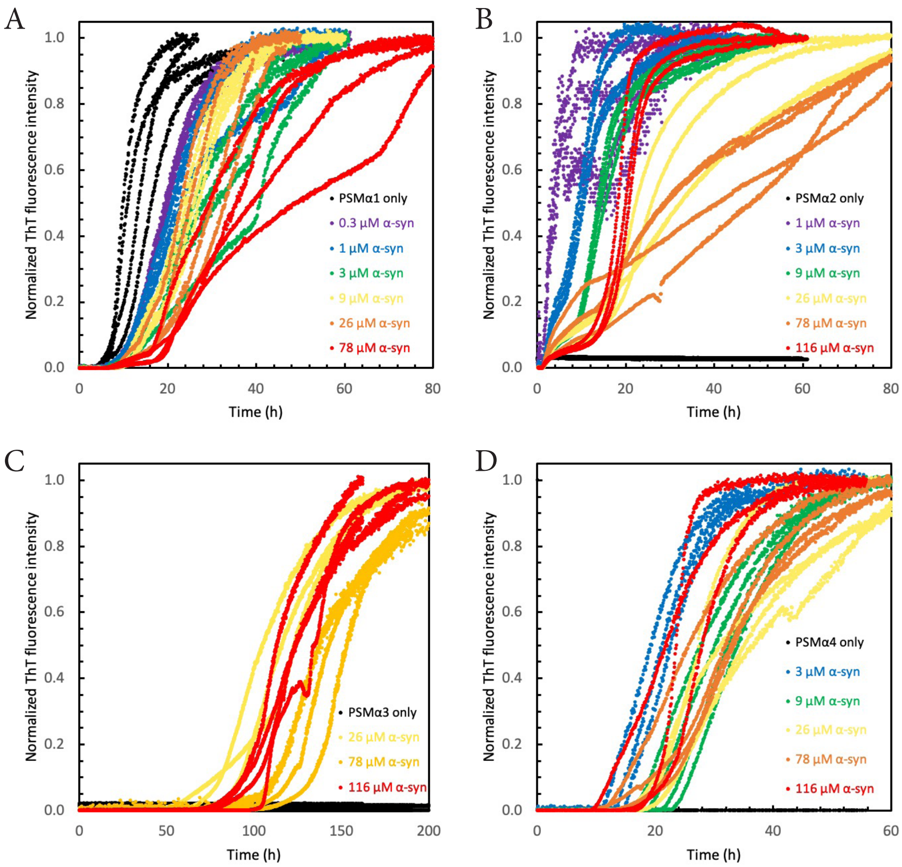


**Supplementary Figure S10. Aggregation of PSMα peptides in the absence and presence of α-syn.** Normalized ThT fluorescence intensity as a function of time for 9 µM PSMα1 (A), PSMα2 (B), PSMα3 (C) or PSMα4 (D) with varying α-syn concentrations as given in the respective panels. All experiments started from monomers and were performed in 10 mM Tris, 50 mM NaCl, pH 7.6 at 37 °C under quiescent conditions.

**Supplementary Figure S11. Electron micro-scopic images.** TEM micrographs at 60000 times magnification of samples taken at the plateau of aggregation reactions of 78 µM PSMα1 (A), PSMα2 (C), PSMα3
(E), PSMα4 (G) peptides with 8.9% DMSO or of 25 µM α-syn in the absence (B) or presence of 9 µM (D) PSMα2 (F) PSMα3 or (H) PSMα4 peptides and 1.1% DMSO. All experiments started from monomers and were performed in 10 mM Tris, 50 mM NaCl, pH 7.6 at 37 °C under quiescent conditions. After deposition on the grids, the samples were labelled by an anti-α-syn primary antibody (syn211) and a secondary antibody linked to 10 nm gold nanoparticles. Scale bar = 200 nm

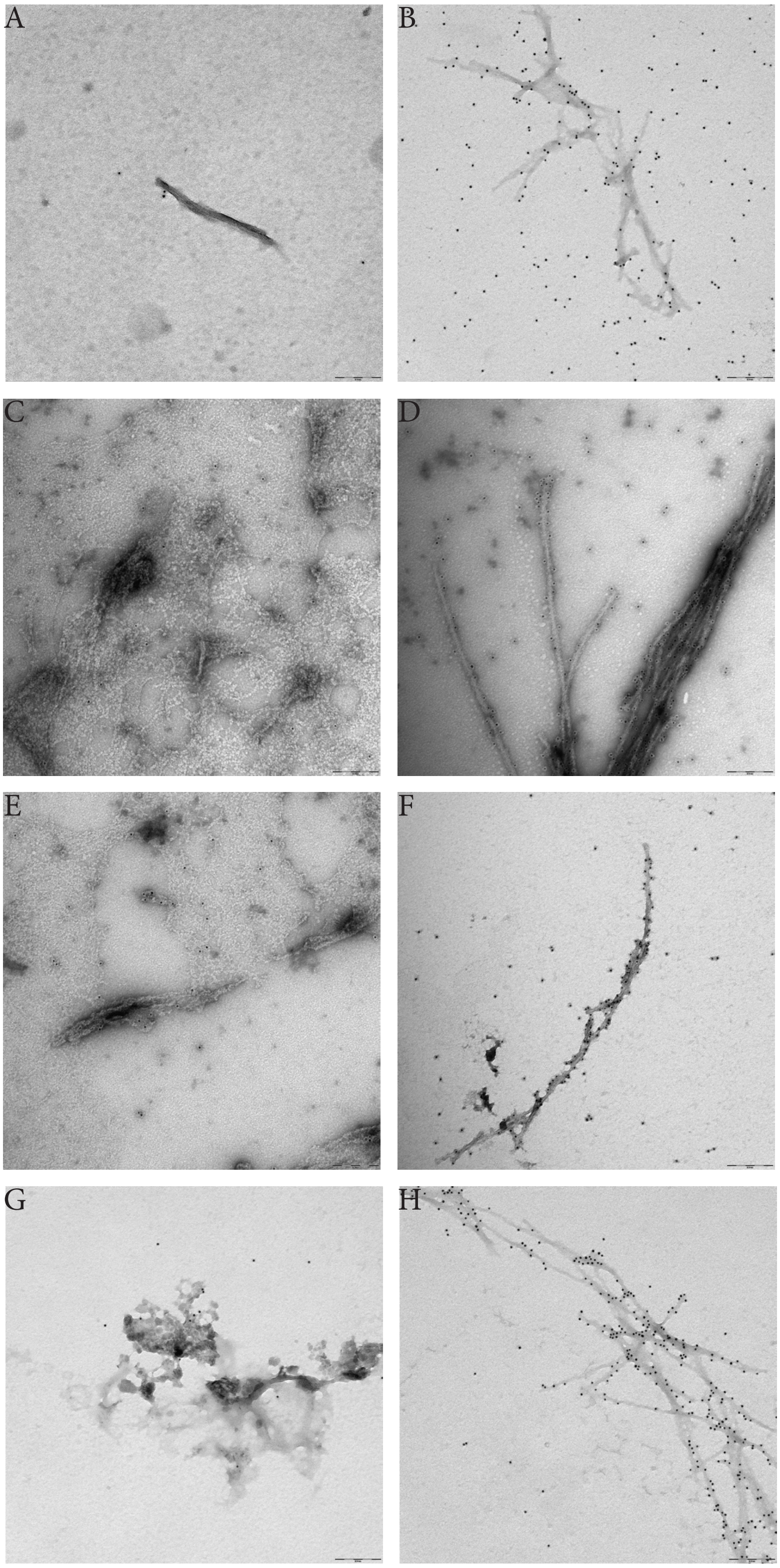

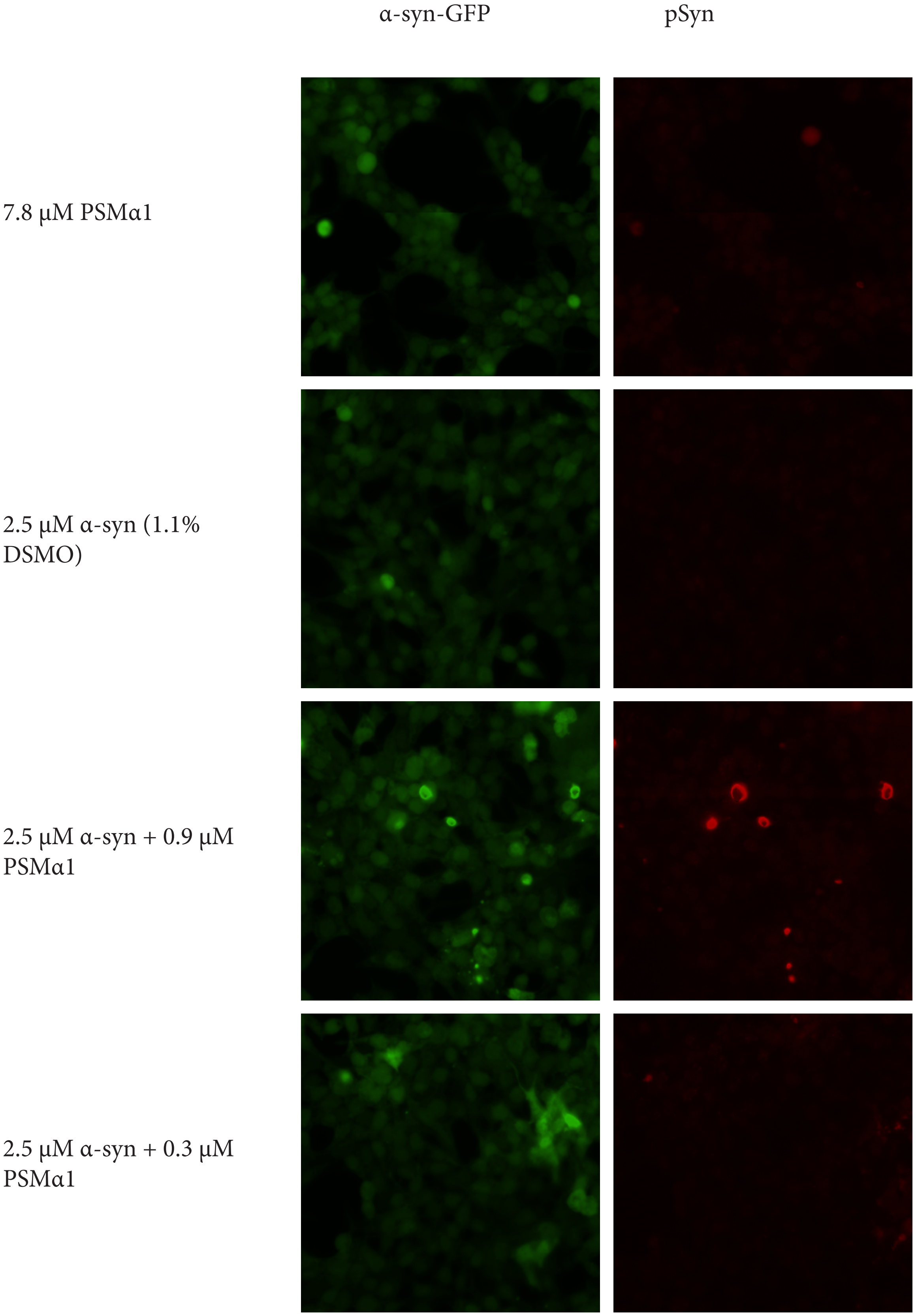
α-syn-GFP
pSyn
7.8 μM PSMα1
2.5 μM α-syn (1.1% DSMO)
2.5 μM α-syn + 0.9 μM PSMα1
2.5 μM α-syn + 0.3 μM PSMα1

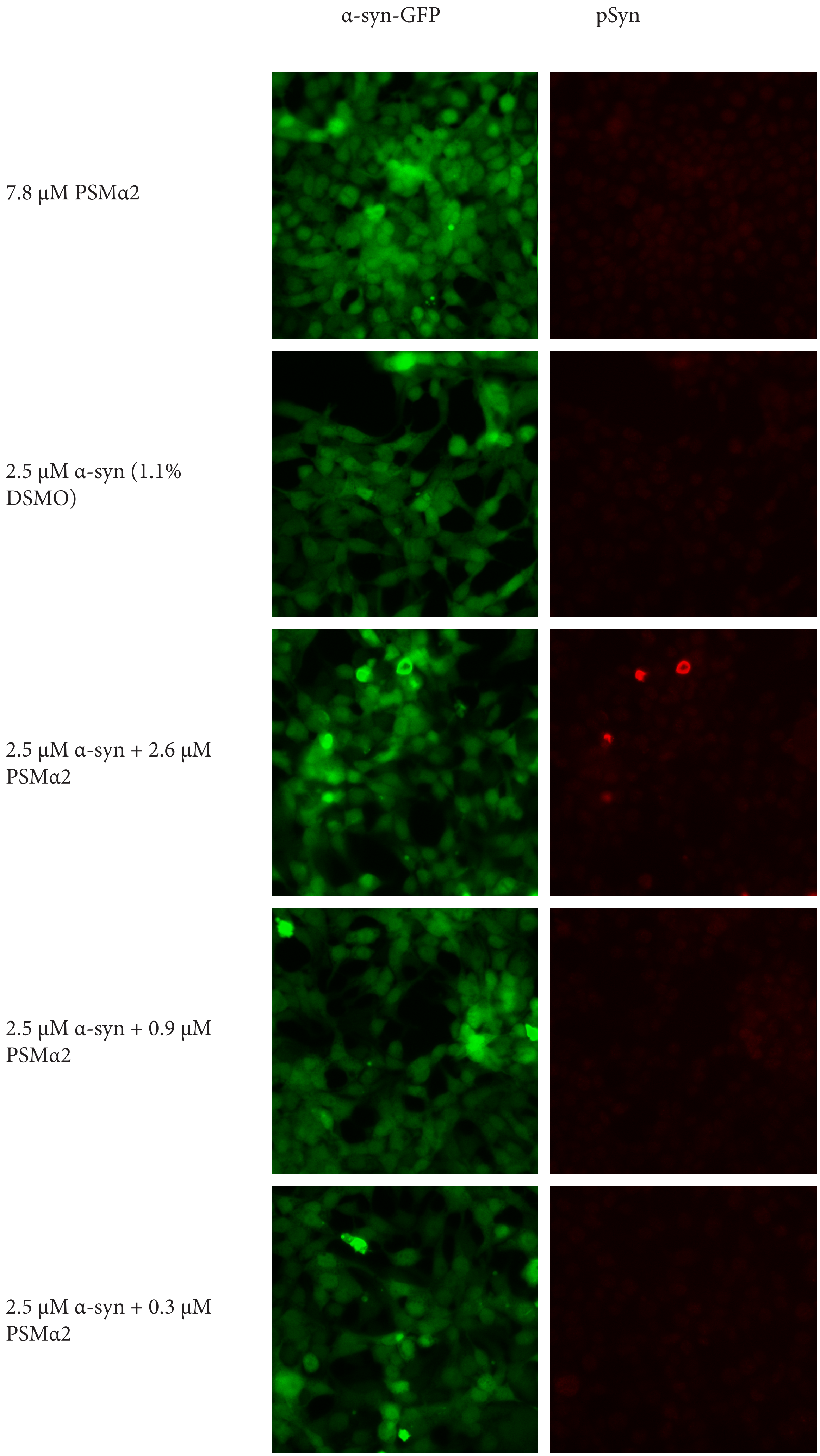
α-syn-GFP
pSyn
7.8 μM PSMα2
2.5 μM α-syn (1.1% DSMO)
2.5 μM α-syn + 2.6 μM PSMα2
2.5 μM α-syn + 0.9 μM PSMα2
2.5 μM α-syn + 0.3 μM PSMα2

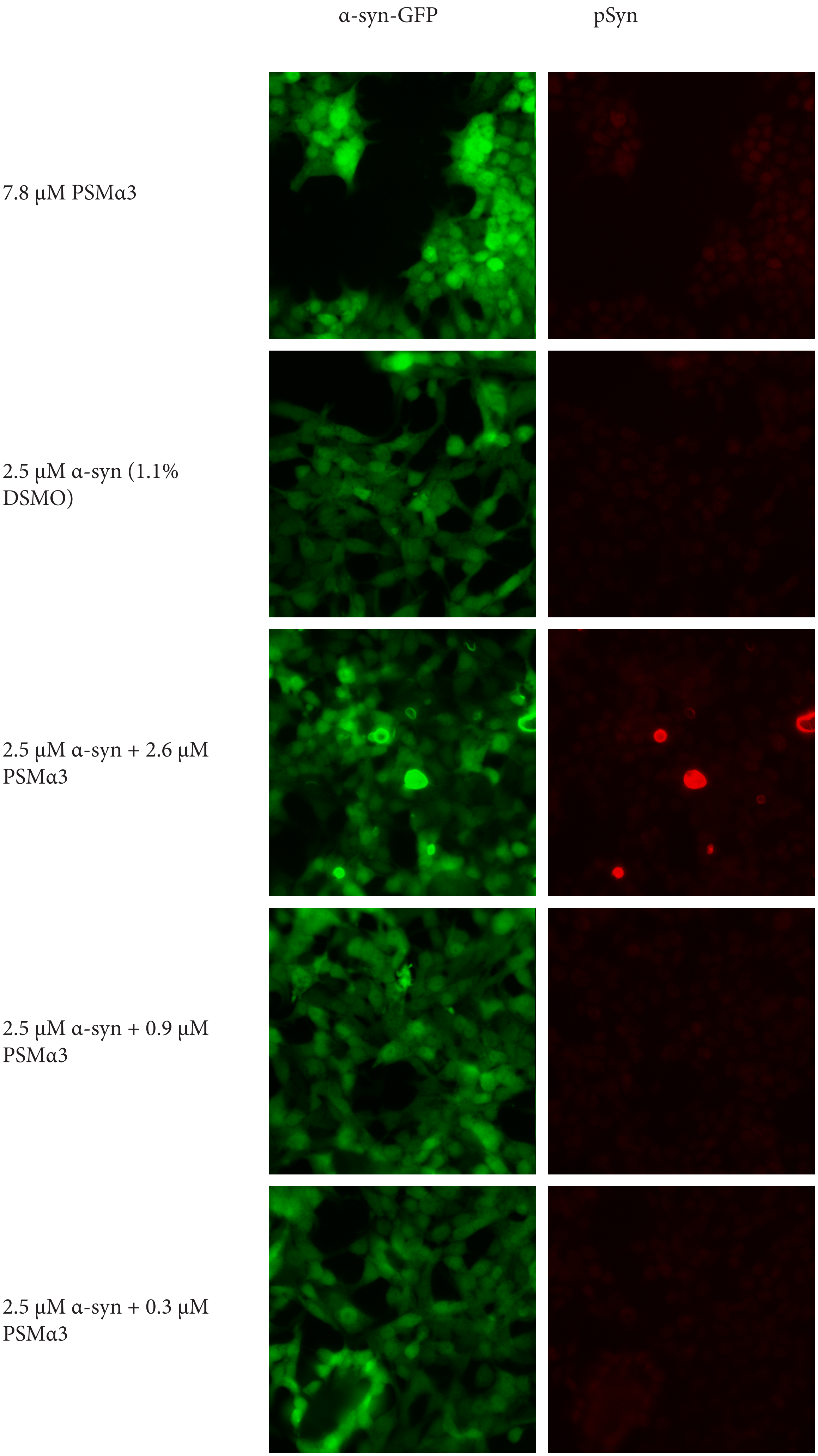
α-syn-GFP
pSyn
7.8 μM PSMα3
2.5 μM α-syn (1.1% DSMO)
2.5 μM α-syn + 2.6 μM PSMα3
2.5 μM α-syn + 0.9 μM PSMα3
2.5 μM α-syn + 0.3 μM PSMα3

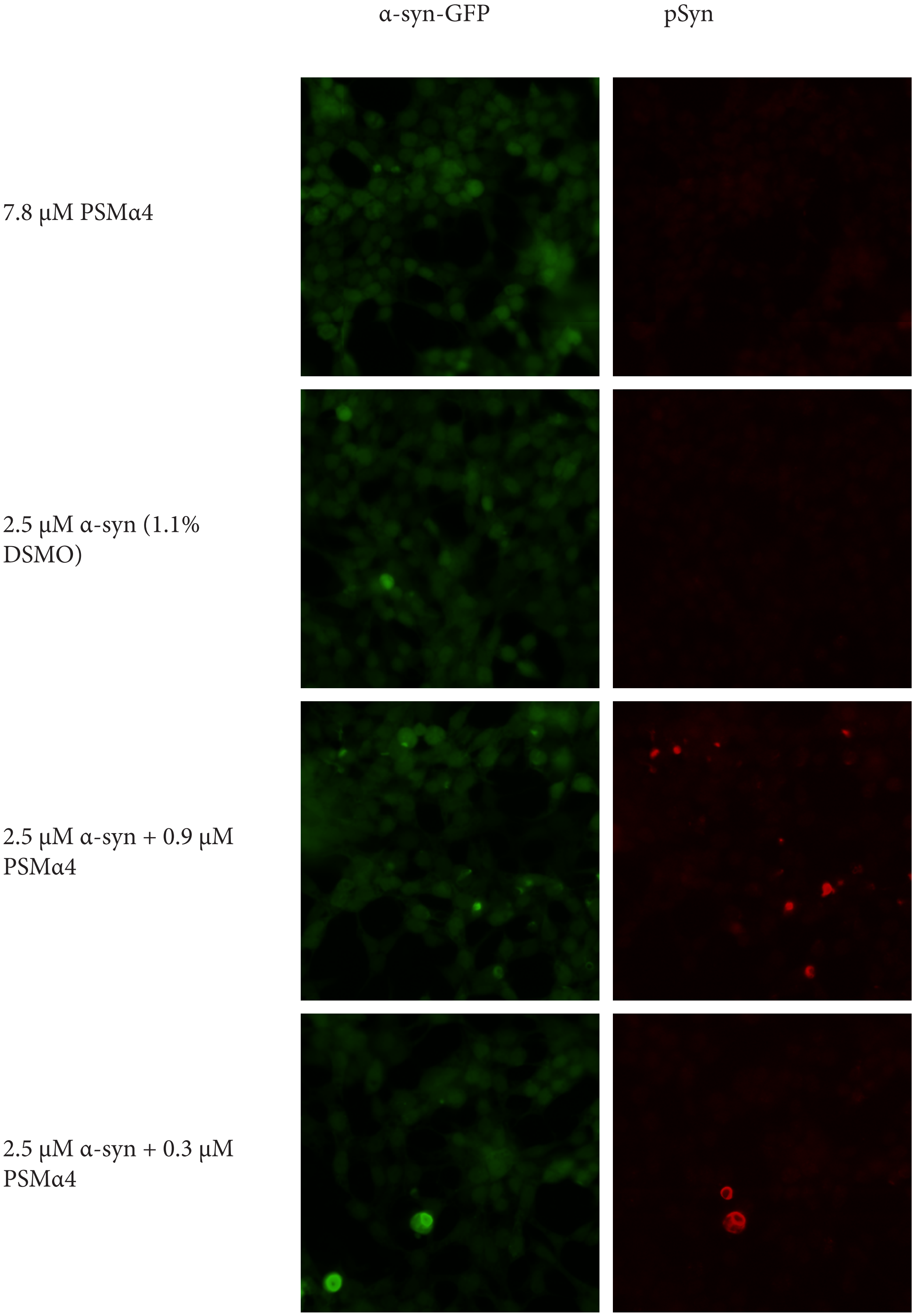
α-syn-GFP
pSyn
7.8 μM PSMα4
2.5 μM α-syn (1.1% DSMO)
2.5 μM α-syn + 0.9 μM PSMα4
2.5 μM α-syn + 0.3 μM PSMα4

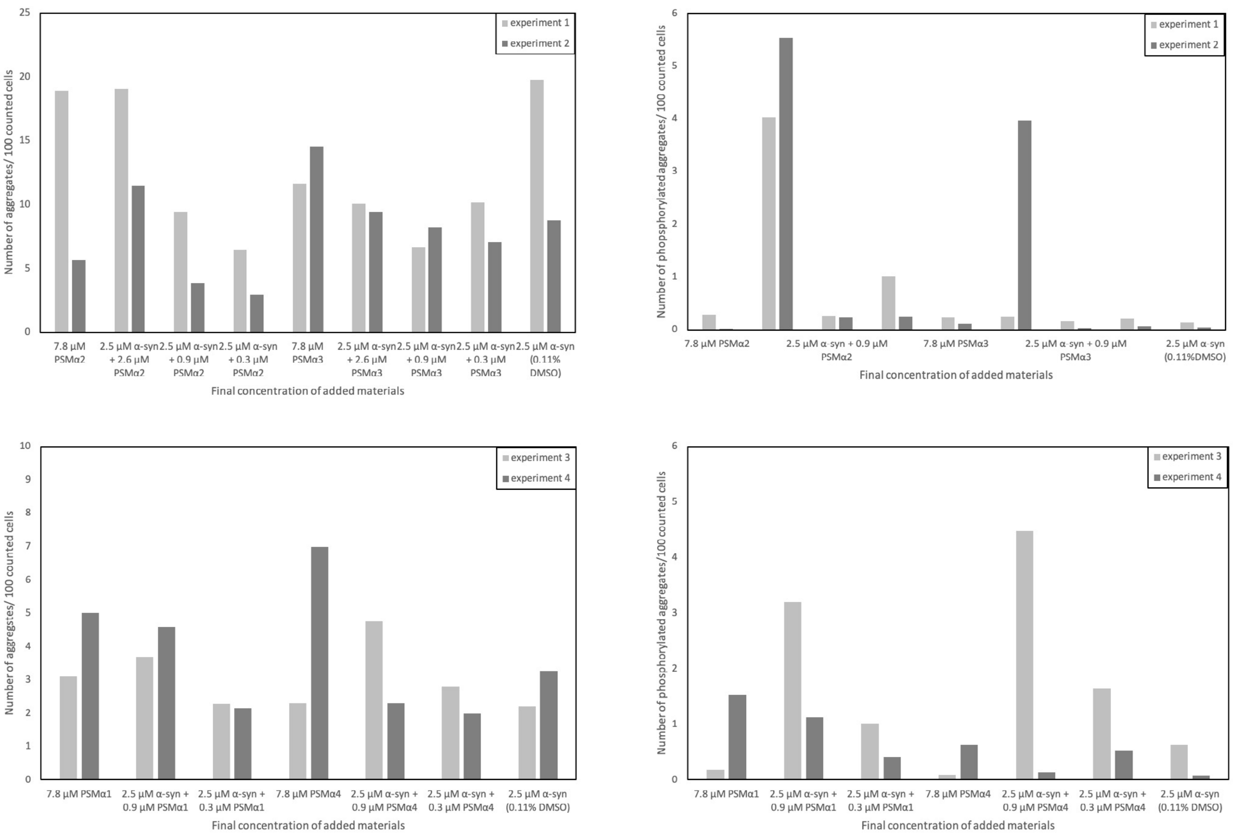


**Supplementary Figure S12. Seeding in HEK A53T-GFP cells.** HEK cells were stained for α-syn phosphorylated at ser129 and a Cy3-conjugated secondary antibody after 48 hour-treatment with α-syn aggregated in the absence or presence of PSMα peptides at different concentrations. Representative images from one well from each treatment condition are shown. These experiments were repeated twice. The number of aggre-gates and phosphorylated aggregates were quantified per 100 counted cells. The averages from all replicate wells (3-4/experiment) are shown for each experiment. The representative images shown are from experi-ments 2 and 3.
